# New Perspectives of Deep Brain Stimulation Indications for Parkinson’s Disease: A Critical Review

**DOI:** 10.3390/brainsci14070638

**Published:** 2024-06-26

**Authors:** Renata Montes Garcia Barbosa, Miriam Carvalho Soares, Denise Maria Meneses Cury Portela, Thiago Gonçalves Guimarães, Rubens Gisbert Cury

**Affiliations:** 1 Movement Disorders Center, Department of Neurology, School of Medicine, University of São Paulo, São Paulo 05403-010, Brazil; renatamontesgarcia@hotmail.com (R.M.G.B.); miriamcarvalhosoares@icloud.com (M.C.S.); thiago.guimaraes@hc.fm.usp.br (T.G.G.); 2 Movement Disorders Center, Department of Neurology, School of Medicine, Centro Universitário Uninovafapi (UNINOVAFAPI), Teresina 64073505, Brazil; denisecury77@hotmail.com

**Keywords:** deep brain stimulation, Parkinson’s disease, genetics, imaging

## Abstract

Deep Brain Stimulation (DBS) is an effective treatment option for patients with dopaminergic complications of Parkinson’s disease (PD) and drug-refractory PD tremor. However, DBS and its indications can be challenging, and they are not often debated in the medical community. Through a critical narrative review, the objective of this paper is to improve the comprehension of DBS indications and help to solve the puzzle that this process can be. Proper patient selection is the first step for a good surgical outcome. In this review, then, relevant considerations are discussed, involving PD genes, PD phenotypes, indications of early stages, non-motor symptoms, neuroimaging predictors, comorbidities, and age. Individualized approaches are encouraged, including clinical and radiological factors. Social support during the whole follow-up and expectations alignment are necessary through this process and are also debated.

## 1. Introduction

Deep Brain Stimulation (DBS) is a recognized evidence-based therapy for the treatment of dopaminergic complications in Parkinson’s disease (PD). The most commonly used DBS targets are subthalamic nucleus (STN) and globus pallidus internus (GPi). DBS introduction and its refinements offer hope for millions of patients with pharmacologically uncontrollable motor fluctuations, levodopa-induced dyskinesias, and drug-refractory PD tremor [1,2]. Hence, DBS has been increasingly included in several medical society guidelines [3,4].

DBS can substantially improve cardinal motor features in the first five years after surgery [5]. In addition, there is evidence that DBS can control levodopa-related motor complications for ten years or longer [5]. Studies comparing STN-DBS with GPi-DBS have shown sustained improvement in motor features (fluctuations, dyskinesias, and on- and off-medication motor function), as well as activities of daily living (ADL) scores at 36 months with both stimulation targets. STN-DBS has also demonstrated a sustained reduction in the levodopa equivalent daily dose (LEDD) [2,6].

There is also financial benefit from this technology. PD has a high economic burden from all perspectives, including patients, caregivers, health insurance, and society. The costs of this illness significantly increase in later disease stages, and both direct and indirect costs contribute to the high burden of advanced PD. Reduction in pharmacologic need is also reflected in decreased costs [7]. Furthermore, as the disease advances, treatment alternatives tend to fail, leading to a worse quality of life. Nevertheless, as the disease progresses, it is likely that the cost-effectiveness of DBS will further decrease over a longer time span, as demonstrated in prospective studies [8].

It is paramount to improve DBS comprehension as it is an outstanding option for PD treatment. The indications for DBS can be compared with a puzzle-solving process. Questions of better clinical predictors for surgery improvement are constantly arising. The levodopa challenge remains a notably useful predictor of DBS outcome. However, recent analysis has shown that although there is a significant correlation between the absolute Unified Parkinson’s Disease Rating Scale (UPDRS)-III reduction during levodopa challenge and DBS clinical off-med response, it does not allow one to predict accurately an individual patient’s improvement [9]. Evidence suggests that preoperative disease severity may be a more important factor for stimulation improvement than the levodopa challenge scores alone [9].

Therefore, as there is no test that definitively predicts a good outcome after DBS, in order to help neurologists in solving doubts when recommending this treatment and putting the “DBS indication puzzle” together, this review will discuss the “best patient profile for DBS surgery”. The focus will be on new relevant considerations that have recently emerged, such as the role and impact of PD genes, PD phenotypes, early stages, non-motor symptoms, neuroimaging, comorbidities, and age, regarding their influence on indication and DBS outcomes.

## 2. Methods

This study was a critical narrative review performed through a comprehensive analysis of the literature. Studies were identified by searching electronic databases and scanning the reference lists of articles. Articles in English published between 1 March 2003 and 13 November 2023 and a total of 79 articles were included.

This was performed by searching the Medline, Embase, LILACS, and Google Scholar databases. The reference and citation list of relevant studies were manually screened for potentially eligible articles. We searched for the terms “Parkinson’s disease”, “Genetics”, “Phenotypes”, “Non-motor symptoms”, “Neuroimaging”, “Comorbidities”, “Age” and “expectations” in combination with “Deep Brain Stimulation”, “subthalamic nucleus” and “globus pallidus internus”.

We analyzed and organized data into the following topics. Choosing the puzzle: the most used criteria to select DBS candidates, showing the indications and prerequisites; Sorting and grouping pieces: what additional information we can use to refine patient selection; Be patient: Is it worth it?: the alignment of the patient’s expectations with the professionals involved in patient selection, the importance of patient autonomy and a discussion about the social support available for the patient.

## 3. Choosing the Puzzle

In 1999, the Core Assessment Program for Surgical Interventional Therapies in Parkinson’s Disease’ (CAPSIT-PD) was published, and the indications provided in that study were widely introduced in DBS centers worldwide, being highly useful in supporting the selection of candidates. Despite the importance of CAPSIT-PD, only 1.6% of patients with PD are eligible for DBS, increasing to 4.5% when applying more flexible criteria [10]. In the following paragraphs, updated indications for DBS in PD will be discussed. Two subitems will be derived from the main topic: “indications” and “prerequisites”. To fulfill the criteria for a good DBS candidate, patients must have at least one indication (as demonstrated in Table 1) and all five prerequisites (as demonstrated in Table 2).

## 4. Sorting and Grouping Pieces

### 4.1. Genetics

The clinical outcomes can differ depending on the genetics (as demonstrated in Table 3), which has already been reported in autosomal dominant PD (SNCA, LRRK2, and VPS35) and autosomal recessive PD (PRKN, PINK1). To date, no cases of DJ-1-PD-DBS have been reported. Furthermore, heterozygous GBA mutations, which are considered to increase the risk and modify the PD phenotype, have been described. Mutations in PRKN, LRRK2, and GBA account for up to 29% of patients undergoing DBS surgery [19].

Other genes and mutations hree cases of 22q11.2 (2 GPi and 1 STN) were reported to have motor improvements between 30 and 70% in the first year and expression improved by 67% [22]. There are also reports of DBS treatment in ATP13A2 (p.R449Q and two Parkin variants) with favorable responses; PLA2G6 (GPi and Ventral intermediate nucleus) control of dystonic storm and tremors; FBX07 (GPi) good response; DNAJC6 (STN), with marked response; and VPS13C (STN) with initial benefits [24].

The reader must bear in mind that most of the studies on the influence of genetics on PD-DBS outcomes are retrospective, anecdotal and short-term. Moreover, most of the studies were focused mainly on a small group of genes responsible for monogenic parkinsonism (PRKN, PINK1, DJ-1, SNCA, LRRKA, and VPS35); hence, it is not possible to state that patients excluded from those studies or included in control groups did not carry pathogenic variants in other genes, as no whole exome or genome sequencing was performed. To further complicate matters, the extent to which polymorphisms and genic modifiers influence the phenotype and DBS response is unknown.

However, the currently available data summarized in the previous paragraphs might help refine DBS indications and guide the neurologist when discussing the pros and cons of the intervention with the patient, particularly in borderline indications.

### 4.2. PD Phenotypes: The Role of Heterogeneity for DBS Selection

Clinical heterogeneity is well-recognized in PD, and several studies have attempted to divide PD into subtypes [25,26]. To define profiles for optimal DBS candidates that encompass the complexity of the PD clinical spectrum, some studies have assessed responses to DBS in relation to these subtypes. In a large longitudinal cohort of PD patients undergoing bilateral STN-DBS, the tremor-dominant (TD) phenotype was a positive predictive factor of short-term motor outcome after surgery. These results are in line with those of a previous study that analyzed the influence of motor phenotype on STN and GPI DBS motor outcomes at 24 months. Significant differences in the responsiveness of PD patients to DBS based on their motor subtype were found, with postural instability/gait disturbance (PIDG) patients receiving less benefit from stimulation than TD and indeterminate subtypes [27].

Additionally, a multicenter study compared a group of patients with a predominance of symptoms on the right (RDP) in relation to another group with a predominance of symptoms on the left (LPD). Patients with LPD presented significantly higher motor and overall NMS impairment. It was observed that motor symptom laterality seems to carry an impact on PD clinical manifestations. It is possible that the laterality of the symptoms may, therefore, influence the benefit of DBS, requiring further studies focused on this subject [28].

### 4.3. Early-Stage Indications: Is There a Haste Enemy of Perfection?

DBS is a well-established adjunctive treatment for patients with moderate–advanced-stage PD. Nevertheless, evidence also supports earlier indications of DBS in PD rather than waiting until medical treatment benefits are lost [29].

Within that scope, a paradigm shift was provided by the Earlystim trial in 2013. The efficacy and safety of DBS were demonstrated in a subgroup of patients with more than four years of PD symptoms and less than three years of uncontrolled motor complications [30]. This led the U.S. Food and Drug Administration (FDA) to extend DBS indications in 2015 to shorter disease durations (from five to four years). The concerns for DBS indications should consider procedural safety, efficacy, and patient preferences. The risks of brain surgery while functional in life activities and additional costs for pulse generator replacements are also concerns for early-stage indications [31].

How early the term “earlier” means is also debated. The recent European Academy of Neurology/Movement Disorder Society (EAN-MDS) 2022 Guideline for PD Treatment labeled “early fluctuations” complications with less than three years of onset. The same document suggests that DBS can be considered in patients with early fluctuations who fulfill the other inclusion and exclusion criteria for DBS [32]. Less is known about the long-term course of PD in patients with early fluctuations than in patients with advanced disease. Another important theme of discussion is the target choice, as Earlystim data were about STN-DBS, and different targets (such as GPi) are not interchangeable [32]. Early-stage surgeries may provide even greater long-term medication reduction than standard care. Hacker et al. demonstrated that the best medical treatment group was five times more likely to have higher PD medication costs than the early DBS patient’s treatment [33]. Another significant aspect to consider is that the reduction of dopaminergic drugs is associated with the amelioration of impulsive-compulsive behaviors.

Earlier STN-DBS surgeries were also ratified by considering the dopaminergic sensitization process. It refers to incremental motor and behavioral responses to a single dose of levodopa after repeated and chronic administration. In degenerative PD, chronic pulsatile exposure to levodopa or dopaminergic agonists leads to a wide spectrum of progressive motor and nonmotor complications. The neuropsychiatric features of dopaminergic sensitization include impulse control disorder (ICD), dopamine dysregulation syndrome (DDS), and neuropsychiatric fluctuations (e.g., on–off related fatigue, anxiety, and inner restlessness). STN-DBS is an option to manage these neuropsychiatric complications, as it allows for medication reduction. Interrupting the dopaminergic sensitization process through medication sparing is important for preventing the development of irreversible clinical and psychosocial issues. Thus, neuropsychiatric issues should not be solely interpreted as contraindications for DBS as it can be alleviated by surgery [34]. Together, these aspects demonstrate the clinical and cost benefits of earlier DBS procedures [10].

Nevertheless, it is important to emphasize that the “earliest time” refers to the moment after the onset of the first dopaminergic complication. This is different from “early indications after PD diagnosis,” with less than the established four years from disease onset. To date, STN-DBS has not been indicated for PD in the absence of dopaminergic complications.

### 4.4. Non-Motor Symptoms Matter

Although there is no precise recommendation on how to consider the presence of NMSs in the selection of PD candidates for DBS, some evidence shows that these aspects could be influenced by this therapy [10]. In a cohort study that investigated bilateral STN-DBS motor, non-motor, and quality of life effects in 60 patients with PD, approximately 40% of the patients treated with DBS improved their NMSs [35].

Few studies have demonstrated the improvement of different NMSs (cardiovascular, sleep/fatigue, perceptual problems/hallucinations, gastrointestinal, urinary, and miscellaneous domains) six months after surgery [36]. For the sleep/fatigue, urinary, and miscellaneous domains, the benefit was maintained at 24 months [35] and for the sleep domain at 36 months [37].

Fatigue and sleep: STN-DBS can modulate sleep physiology via direct effects on the STN or a spread of electric current to projections from regions in proximity to the STN, such as the pedunculopontine nucleus (PPN), thus resulting in an improvement in sleep architecture [38]. A prospective study showed that fatigue, as assessed using the MDS-UPDRS, significantly improved at the six-month evaluation [38,39]. Another multicenter study was the first to report significant beneficial effects on fatigue at 24-months follow-up [35]. The same study demonstrated a significant subjective improvement in sleep disturbance at the 24-month follow-up and was the first to report significant beneficial effects of STN-DBS on daytime sleepiness in contrast with previous studies [35,40,41].

Pain: Pain in patients with PD is a complex and increasingly recognized non-motor symptom [42,43]. It has been classified into five main categories: musculoskeletal pain, radicular or neuropathic pain, dystonia-related pain, akathisia discomfort, and primary or central Parkinsonian pain [43]. The improvement in global pain scores after STN DBS ranged from 28% to 84% compared with the preoperative baseline. Better control of motor symptoms by STN-DBS might improve fluctuation-related and dystonic pain [43]. A prospective study conducted in France demonstrated a remarkable decrease in pain fluctuations after chronic stimulation [42].

Cognition: The strongest predictors of cognitive impairment found in the three largest sample studies regarding DBS for PD were older age, higher LEDD, poorer levodopa response, freezing of gait, and attention/executive impairment [44,45]. A kinetic-rigid phenotype is also widely recognized as a risk factor for dementia [46]. An interesting point of discussion is how cognition influences the preoperative selection of patients. Few studies have investigated the role of preoperative cognitive burden in short-term motor changes after surgery. In a large longitudinal cohort of PD patients with bilateral STN-DBS the white matter hyperintensities of vascular origin (WMHs) on preoperative brain MRI were a predictor of worse long-term motor outcomes. A strong association between cognitive and axial impairments, WMH signal burden, and perivascular spaces in the basal ganglia has been found in patients with PD. White matter ischemic lesions are associated with earlier onset of PD, higher severity of cognitive impairment, and the PIGD phenotype. This can be related to diffuse ischemic damage involving subcortical non-dopaminergic pathways. This observation highlights that preoperative brain imaging is another important variable in patient selection, as will be shown below [27].

However, regarding DBS-induced cognitive dysfunction, a recent systematic review showed evidence of a deterioration in verbal fluency. In this study, the impact of DBS on memory, attention, executive function, and processing speed was inconclusive. Furthermore, global cognition does not appear to be affected by DBS [47].

Impulse Control Disorders: Impulse control disorders: The effect of chronic STN-DBS on impulse control disorders (ICDs) and dopamine dysregulation syndrome (DDS) has been estimated in several studies [34]. Dopamine agonist dose reduction was the main driver of ICD improvement, as demonstrated in a study that showed an improvement of 95% in patients with preoperative ICD [48,49]. In contrast, STN-DBS could lead to an increase in impulsivity caused by the estimation of non-motor parts of the STN. This situation can be reversed by DBS or medication adjustment [34]. A secondary analysis of the Earlystim trial also demonstrated that patients with preoperative hyperdopaminergic behavior did not have an increased risk of worsening behavior with subthalamic stimulation compared with medical therapy alone [50].

Nevertheless, some important considerations are necessary to determine the best instrument to access the NMS during DBS selection of PD patients. The Ardouin Scale of Behavior in Parkinson’s Disease (PD) is a well-known instrument. The presence of ICD was defined as at least two scores ≥ 2 or at least one score ≥ 3 (severe ICD). In such situations, a sensible strategy would be to address ICD before surgery. For instance, by reducing or discontinuing dopaminergic agonists and being attentive to apathy [27].

Despite available evidence showing improvement in some NMS after DBS, this surgery might not be the best therapeutic option to consider if the disease burden is mainly driven by non-motor symptoms. Studies on treatment responses of specific NMS subtypes to different treatment strategies are required and may help to provide individualized medicine for patients’ real-life requirements [36].

### 4.5. Neuroimaging: How Can Neuroimaging Contribute to DBS Indications?

Preoperative screening by neuroimaging, in particular by brain magnetic resonance imaging (MRI), allows for the identification of structural lesions that may increase surgery risks, investigate evidence of atypical parkinsonism, and influence surgery planning. Preoperative MRI can also impact DBS indications, as evidence suggests that patients with great microvascular lesions may have lower benefits from surgery [51].

Excessive cortical atrophy increases the risk of postoperative subdural hematomas. Brain atrophy also accounts for some variability in DBS outcomes [51,52]. Brain atrophy occurs in patients with PD and affects various cortical and subcortical structures, including the lateral ventricles, sensorimotor, parietal lobe, perisylvian cortex, hippocampus, and caudate nuclei. The sensorimotor cortex is among the most affected areas by PD atrophy and is directly related to increased motor symptom severity [52].

A retrospective cohort of patients with STN-DBS demonstrated that presurgical thalamic and ventricular volumes predicted the degree of motor score improvement after DBS [52]. Technically, increased ventricular size may contribute to surgical targeting’s difficulty in reaching the STN and may predispose patients to electrode shift. Additionally, the thalamus is typically penetrated by the electrodes’ route to the STN, making this a possible contributor to the structural causes of inaccuracy. As the ventroanterior and ventrolateral nuclei of the thalamus are major downstream outputs of the GPi and STN, structural thalamic changes may hypothetically affect functional pathways by which DBS exerts its therapeutic effects [52].

Since DBS not only changes the local neural activity in the nuclei but also the fiber tracts near the stimulation site, targeted cerebral networks could be predictors of postoperative clinical response. The cortical integrity of the frontal regions may also have a role in DBS outcomes. The integrity of the frontal cortex (measured by analysis of the paracentral area and superior frontal region cortical thickness) can predict the effects of STN-DBS in patients with PD [53].

Furthermore, the hypothesis that the effectiveness of DBS in PD is related to connectivity dysfunction between the stimulation site and other brain regions is growing [54]. A specific study examined functional connectivity between the STN and other brain regions in patients with PD who were candidates for DBS together with cases in similar stages but not candidates for DBS. Decreased functional connectivity was observed between the STN and sensorimotor cortex in patients eligible for surgery relative to non-candidates for neurostimulation, and motor sign severity was correlated with this effect [54]. Thus, alternative approaches for evaluating functional connectivity (FC) in DBS patients may improve the analysis of its effects [55]. Evidence also suggests that preoperative STN-GPi FC predicts DBS-related benefits for motor PD symptoms. Thus, FC may be a promising biomarker of DBS responsiveness [55].

Neuropsychological decline after DBS surgery occurs in approximately 10–15% of patients. Imaging with iron deposition measurements (susceptibility MRI sequencing: *R2) is increasingly recognized as a neuropsychological outcome predictor after DBS surgery. Iron is directly related to pathological processes and the progression of PD, including Lewy body catalyzation and aggregation [56]. Specifically, iron deposition measured by *R2 imaging of the substantia nigra, caudate, STN, putamen, and hippocampus was related to executive and attention performance outcomes after DBS [56].

### 4.6. Comorbidities

A higher number of clinical comorbidities is a consistent risk factor for complications in widely performed procedures [57]. Concurrent diseases are also positively correlated with readmission rates within 30 days of neurosurgeries [58]. Attention should be direct towards cardiovascular risk factors such as hypertension, hyperlipidemia, diabetes, coronary artery disease (CAD), obesity, and smoking. The literature infers a cumulative effect of medical comorbidities, with a higher number of clinical issues positively related to increased mortality and readmission rates after DBS surgery [59]. The literature supports that one of the strongest predictors of complications in DBS surgeries is the number of comorbidities [60]. The rate of DBS readmission ranges from 1.9 to 4.3% within 30 and 90 days, respectively [61]. Surgery-related issues are the most common cause of complications and unplanned readmission. Infections in postoperative care have an estimated incidence of 4.5–5.6% in DBS surgeries. In summary, DBS is a safe procedure with 30-day readmission rates lower than those of general neurosurgeries and other commonly performed procedures [61,62].

The co-occurrence of cardiac arrhythmias in patients with PD raises doubts about the interferences and security of DBS surgery in patients with permanent cardiac pacemakers (PPM). The case series demonstrated no serious adverse events in patients who underwent DBS surgery with a previously implanted PPM [63]. Suggestions to mitigate interferences are mentioned, such as DBS IPG placement at different sites from the PPM; prioritizing DBS parameters to bipolar stimulation; when possible, adjustments of PPM signal filtration; and a close follow-up of both cardiology and neurology teams. As there is no prospective study and no robust evidence in this regard, DBS indication in patients with PPM should be carefully analyzed.

Other chronic conditions related to readmission rates after DBS surgery include acquired immunodeficiency syndrome (AIDS), alcohol abuse, autoimmune disorders, peripheral vascular disease, and renal failure [60]. The impact of infectious comorbidities, such as human immunodeficiency virus (HIV), in patients with sustained virological control has been recently evaluated. A small cohort of nine patients with PD and HIV demonstrated no serious adverse events after DBS surgery and significant improvement in motor scores was verified, which persisted during the five years of follow-up [64].

### 4.7. Deep Brain Stimulation in Elderly

Age is a controversial factor in DBS patient selection. PD centers diverge in using different cut-offs or no-cut-off of age [65]. The same applies to clinical trials, where some studies considered the age of 75 years or 80 years as a threshold, while other centers had no specified maximum age for studies [66].

Age-related changes modify anatomy, possibly reducing the relative distance between the intended target of stimulation and surrounding structures. Other neurodegenerative co-pathologies, such as Alzheimer’s disease and higher comorbidities burden may also be present and can impact surgery outcomes [65].

Several studies comparing younger and older patients showed improvement in QoL, motor UPDRS, and total UPDRS [66,67,68]. In two of these studies, QoL improved after DBS in elderly PD patients, but this improvement was not as sustained as that observed in young patients with PD. Another issue to consider is that in those studies, the group of 70-year-olds and the older group had a lower effect size in comparison to the youngest samples [66,67,68].

The literature has also shown that a significant reduction in LEDD has been observed in young and old patients. Both groups showed similar reductions in motor complications [67]. In a study of 27 patients, the LEDD was reduced from 650 mg (baseline) to 280 mg at 1 year and 325 mg at the final follow-up (between 21 and 108 months) [66]. These findings suggest that STN-DBS can reduce LEDD in elderly patients, especially in those taking large doses of levodopa, with accompanying complications.

Another study comparing patients aged >65 years showed slightly higher incidences of postoperative confusion/psychosis in elderly patients [67]. Vesper et al. also divided patients into these two age groups (<65 years and ≥65 years), in that study infection rates were significantly more frequent in the older age group than in the younger ones. Dementia incidence during the 2-year follow-up did not differ between the older and younger patients, which was also similar to the overall cognitive impairment found in PD natural history [69].

Surgical procedures in patients of advanced age (especially over 70 years) should be judiciously evaluated, as the risk/benefit ratio becomes less favorable [70]. Therefore, cumulative comorbidities and cognitive burden should be analyzed extensively. A broad comprehension of biological age should be considered, rather than a simple numerical threshold [57,59].

Additionally, the technological advancements of DBS, like an improved implanted pulse generator (IPG) design, can help reduce the risk of complications in the profile of patients with more comorbidities and advanced age. IPGs are now smaller and round-edged, resulting in a reduced risk of complications such as infections and erosions. Furthermore, it is possible to choose between rechargeable and non-rechargeable options and MRI-compatible devices [71].

## 5. Be Patient: Is It Worth It?

### 5.1. Align Expectations: Patient’s and Physician’s

High-quality clinical decisions regarding medical management should reflect patients’ expectations and objectives and their individual clinical characteristics [72,73]. Realistic expectations of DBS are important for patient selection. Studies have demonstrated that patients with unrealistic expectations, or with suboptimal education on the benefits of DBS prior to surgery, have been reported to more frequently experience postoperative psychological distress and general dissatisfaction with surgery outcomes [74,75].

Yen et al. demonstrated a computer application that allowed patients with PD to describe their symptoms and learn how effectively DBS addresses their prioritized complaints. Additionally, it has been demonstrated that these applications can improve patient knowledge of DBS for PD. Self-directed learning through the app is tied to patient satisfaction after DBS, independent of objective measures on clinical scales [72].

Apathy and depression scores tended to be higher in patients with a negative perception of surgical outcome after STN-DBS surgery, based on an interview that included various domains that may have an impact on a patient’s life with PD and DBS [76]. Although the same patients experienced significant motor improvements, as measured by the UPDRS-III scores, the overall impact of surgery was negative. Therefore, preoperative apathy and depression scores might be helpful tools to identify ‘risk candidates’ for surgery dissatisfaction, providing an opportunity for psychosocial support and counseling [76].

Quality of life (QoL) is an important and largely utilized indicator of treatment results in PD. In a secondary analysis of the Earlystim trial. Patients with worse baseline scores on the Parkinson’s Disease Questionnaire-39 (PDQ-39) scale demonstrated greater postoperative improvement after 24 months of follow up [77].

Age has a complex correlation with QoL. It is important to note that as PD progresses, unresponsive DBS symptoms become more common (e.g., axial, non-motor, and cognitive issues). These symptoms seem to largely impact QoL outcomes, and it is important to align expectations about the likely impossibility of improvement by DBS in these situations.

Few studies have described the long-term measures of QoL in patients with DBS. The literature suggests that the improvement verified in the first 3 years of treatment is followed by a decline in baseline scores over 3–5 years after surgery [2]. This evolution of QoL measures has been observed in both GPi and STN-DBS studies [6].

### 5.2. Patient Autonomy and Social Support

The ethics involved in psychopharmacological neurointerventions raise important questions about how patients’ subjective feelings are significant in the context of DBS outcomes. PD does not affect everyone in the same way and affects many aspects of patients’ daily lives. In that scenario, a recently published study by Chacón Gámez et al. demonstrated that DBS improvement in motor skills is generally translated into better QoL and autonomy for patients and caregivers [78].

Behavioral effects, such as cognitive decline and psychosis, following DBS for PD occur at a low rate, but can change patients’ and caregivers’ lives significantly. In addition, neuropsychiatric symptoms may be associated with higher postoperative risks, such as infection and broken cables. This could be minimized by judicious preoperative neuropsychological assessments. Patient compliance for postoperative consults and neurologist’s recommendations, as well as the maintenance of social support, is also a necessary aspect to be evaluated. Non-compliance with treatment recommendations is associated with reduced benefits and potentially increased harm after DBS surgery [79].

The possibility of being awake during the procedure, the need for a caregiver to help with programming, the requirement for future battery replacements, and a very clear discussion of potential risks are issues that should be extensively discussed in preoperative appointments.

## 6. Conclusions: The Big Picture Perspective

This review summarizes important and complex variables. Solving the puzzle of proper selection of DBS patients is not an easy task. Better selection leads to a greater likelihood of benefits. Improving neurologists’ awareness of precise DBS indications for PD helps in not delaying potentially beneficial procedures and not depriving patients of their treatments. Understanding the influence of age and comorbidities on surgical outcomes helps individualize treatment care. As elderly people and patients with comorbidities may still benefit from DBS, more intense and focused postoperative care can be planned [66].

Genetic factors have gained relevance because they may affect the long-term effects of invasive therapies [20]. Larger cohorts with longer follow-up periods are needed to reliably decipher genotypic differences in DBS outcomes [21]. Assessments of non-motor fluctuations, hyperdopaminergic behaviors, and ICDs are needed and may be better analyzed in future clinical trials. Non-motor symptoms should have greater importance in DBS indication and targeting in the near future [34]. The role of neuroimaging in DBS outcome prediction and management is increasing. Cortical micro structural patterns, functional connectivity (FC), cortical integrity and basal nuclei with iron accumulation seem to correlate with DBS responses [51,52,53].

DBS should not be reserved as a final therapeutic option. Some cases have great benefits from earlier indications, especially in patients whose symptoms significantly impact their QoL [29]. The role of the caregiver during the decision-making process for DBS surgery may also influence and pose other ethical challenges. The ultimate decision to undergo DBS surgery should be the patient’s. Neurologists should be certain that the decision to undergo surgery occurred without coercion.

Furthermore, it is important to provide good social support during follow-up. Good social support is mandatory to ensure pharmacological adherence, rehabilitation program involvement, psychological benefits, and DBS-adjustment guidance.

**Figure 1:**
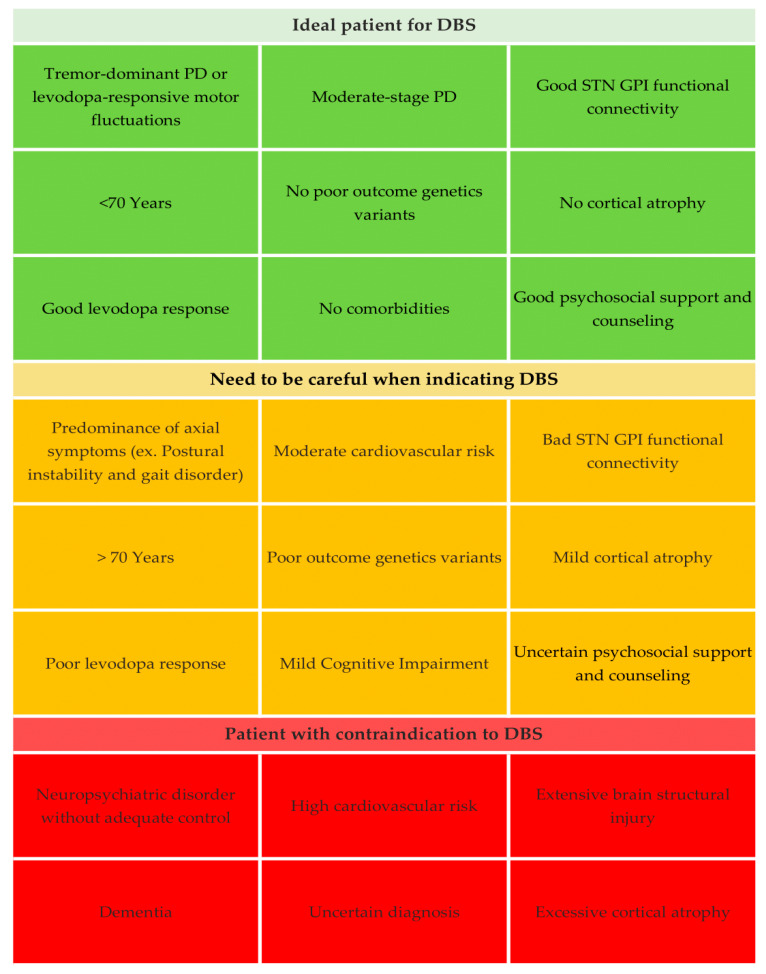
Profile of patients for DBS.

Individualized approaches considering the patient’s characteristics, alignment of expectations, and precise weighting of the individual risk/benefit profile are recommended to resolve this challenging puzzle (Figure 1).

## Figures and Tables

**Table 1:** Summary of the three PD DBS indications.

The Three Indications	Definition	Practical Points
1. Motor Complications	Motor fluctuations and dyskinesia	5-2-1 criteria *
2. Tremor Refractory to Optimized Treatment	Levodopa-resistant tremor [10].	LEDD of ≥900 mg ^β^
3. Intolerance to Dopaminergic Agents	Patients who have adverse effects that prevent the increase in dose to a level that promotes symptom amelioration	Somnolence, hypotension, nausea, vomiting, impulse control disorders and psychosis secondary to dopaminergic medications

* A study to identify advanced PD patients proposed the 5-2-1 criterion as a screening tool: 5 (5 oral levodopa tablets taken per day), 2 (2 h of OFF time per day), and 1 (1 h per day of troublesome dyskinesia) [11,12]. Although this criterion is used for patient selection, it is not mandatory to have prolonged off-time and disabling dyskinesia. ^β^ A proposed definition in an Ultrasound Thalamotomy study of medication-refractory tremor was “a tremor not suppressed by a levodopa equivalent daily dose (LEDD) of ≥900 mg [13]. A study reviewing the data of 149 DBS-DP patients found that the mean maximal LEDD within the medication-refractory tremor group was 851 mg +/− 330 mg [14].

**Table 2:** Sorting the puzzle pieces: the five prerequisites that a good PD DBS candidate must fulfill.

The Five Prerequisites	Why Is It Important?
1. The patient must have Parkinson’s Disease	Atypical parkinsonism does not benefit from DBS [15].
2. More than 4 years after disease onset	This is a measure aimed at avoiding operate atypical parkinsonism * [10,16].
3. Cut-off of 33% in the levodopa challenge test **	The need for the test is supported by the good correlation observed between the percentage of amelioration in the test and after DBS surgery [17].
4. Absence of significant cognitive deficits or uncontrolled neuropsychiatric diseases	Patients with dementia do not benefit, and those with uncontrolled neuropsychiatric diseases have higher risk of complications.
5. Patients must be able to attend frequent medical appointments after surgery	It is paramount that, after the procedure, good programming, medication adjustments when needed, and rehabilitation are performed and prescribed [17].

* Clinical diagnosis is particularly challenging in the early stages. Since Movement Disorders Society (MDS) proposed clinical diagnostic criteria for early PD, the specificity has improved to 95.4% when applied in patients with less than 5 years of disease duration against expert clinical diagnosis. Accordingly, with the improvement in diagnosis, the individualization of the indication for DBS in the early stages will become more viable [18]. ** This test measures the effect of a suprathreshold dose of levodopa by comparing UP DRS-III score off med (12 h withdrawal of dopaminergic medications) and on med (peak of dose) [10,17].

**Table 3:** Most common PD genes and their responses to DBS. AD: autosomal dominant. AR: autosomal recessive. RBD: REM sleep behavior disorder.

Gene	Motor Symptom	Non-Motor Symptom	Good DBS Outcome Variants	Bad Outcome Variants
*LRRK2*-AD	Late-onset PD	Mild or absent	p.G2019S p.G2385R p.T2031S, p.Y1699C p.R793M [19]. *	p. R1441G *
*SNCA*-AD	May have atypical features **	Cognitive decline (70%)	Duplications [19]. ***	p.A53E ^A^
*VPS35*-AD	Similar to tremor-dominant PD	Minimal cognitive, even in the long term	Generally good responses ^α^	-
*PRKN*-AR	Similar to PD. Foot dystonia	Depression	Generally good responses ^£^	-
*PINK1*-AD	Similar to *PRKN*	Some may develop dementia at later stages [20].	Generally good responses ^€^	-
*GBA*-AD	Younger Faster progression More axial symptoms	Dementia, RBD, autonomic dysfunction, and visual hallucination are common and severe	Generally good motor responses ^¥^	GPI-DBS led to a lesser motor improvement of around 22% [20].

* Following surgery, most patients had stable cognitive performance, except for two patients with p.T2031S who developed hallucinations and levodopa dysregulation syndrome 5 years after the procedure [19,20]. ** Atypical features: anterocollis or retrocollis, pyramidal signs and alien limb syndrome [20]. *** Cognitive symptoms seem to remain stable [19]. The nucleotide polymorphisms rs356219 and rs356219, especially when homozygous, may predict a more favorable motor and axial response to DBS [21]. ^A^ patient with p.A53E was wheelchair-bound and demented 3.5 years after device implantation [20]. ^α^ VPS35 DBS outcomes are generally good, with a UPDRS-III change between 36 and 76% [19]. However, data are often incomplete in series and reports [22]. ^£^ The motor outcomes of STN-DBS are good (improvement of 46–84%) and sustained. However, a significant decline in cognitive function has generally not been reported. GPi-DBS led to a UPDRS-III improvement of only 21%, whereas the UPDRS-IV (motor complications) improved by 70% [20,22]. ^€^ Motor improvement with STN-DBS varied from 46 to 62%, and 27% in GPi-DBS [20]. The patient with GPi-DBS had a p.L347P variant and suffered from painful dystonia of the lower limbs and progressive gait dysfunction, which led to the need for walking aids four years after surgery. Similarly, a patient with the p.Arg207* variant showed transient improvement after STN-DBS and developed freezing of gait and dyskinesia 1 year after surgery [20]. ^¥^ Variant p.L444P is more deleterious, with rapid progression to dementia and visual hallucinations. Conversely, more benign variants such as E326K tend to develop dyskinesia. Overall, motor outcomes tend to be quite satisfactory (>50%), similar to those of patients without GBA1 pathogenic variants (non–GBA1-PD) [19,23]. Nevertheless, postoperative cognitive impairment appears to be more common and severe. In one study, their cognition was worse than that of control patients after 7.5 years [19]. A case series of three patients (one p.N370S and two p.L444P) reported cognitive decline after 6–10 years [21]. A recent study comparing the rate of change in cognition between GBA carriers and non-carriers, with and without STN-DBS, concluded that the combined effect of GBA mutations and STN-DBS negatively impacts cognition. Based on the study results, the authors advise considering testing DBS candidates for GBA mutations as part of the presurgical decision-making process. However, most of these results have been assessed only in retrospective studies, and interpreting the results of these studies is limited [23]. In addition, they highlight that although GPi may result in less cognitive decline, no sufficient data are available on the cognitive outcomes of GPi-DBS in GBA-PD patients [23,24]. Therefore, DBS should not be contraindicated based only on the GBA1 status.
